# Supplementary material for: Naturally Available Flavonoid Aglycones as Potential Antiviral Drug Candidates against SARS-CoV-2
Source: Molecules. 2021 Oct 29;26(21):6559. doi: 10.3390/molecules26216559 (PMC8587465; doi:10.3390/molecules26216559)
Supplement: Supplementary file 1 [file molecules-26-06559-s001.zip › molecules-1433028-supplementary.pdf]

Naturally Available Flavonoid Aglycones as Potential Antiviral Drug Candidates against SARS-CoV-2

Ahmed A. Al-Karmalawy[1, ,ψ,*], Mai M. Farid[2,ψ], Ahmed Mostafa[3], Alia Y. Ragheb[2], Sara H. Mahmoud[3], Mahmoud Shehata[3], Noura M. Abo Shama[3], Mohamed GabAllah[3], Gomaa Mostafa Hedeab[4,5], Mona M. Marzouk[2]

[1] Department of Pharmaceutical Medicinal Chemistry, Faculty of Pharmacy, Horus University-Egypt, New Damietta 34518, Egypt. Email: akarmalawy@horus.edu.eg

[2] Department of Phytochemistry and Plant Systematics, National Research Centre, 33 El Bohouth St., Dokki, Giza, Egypt, P. O. 12622. Emails: mainscience2000@gmail.com; aliayassin81@yahoo.com; monakhalil66@hotmail.com

[3] Center of Scientific Excellence for Influenza Virus, Environmental Research Division, National Research Centre, 33 El Bohouth St., Dokki, Giza, Egypt, P. O. 12622. Emails: sarahussein9@yahoo.com; shehata_mmm@hotmail.com; noura.mahrous1995@gmail.com; gaballah09@gmail.com; ahmed_elsayed@daad-alumni.de

[4] Pharmacology Department& Health Research Unit, Medical College, Jouf University, KSA. Email: gomaa@ju.edu.sa

[5] Pharmacology Department, Medical College, Beni-Suef University, Egypt.

*Corresponding author:

Ahmed A. Al-Karmalawy: Email: akarmalawy@horus.edu.eg

ORCID: 0000-0002-8173-6073

ψ These authors equally contributed to this work.

Supplementary data

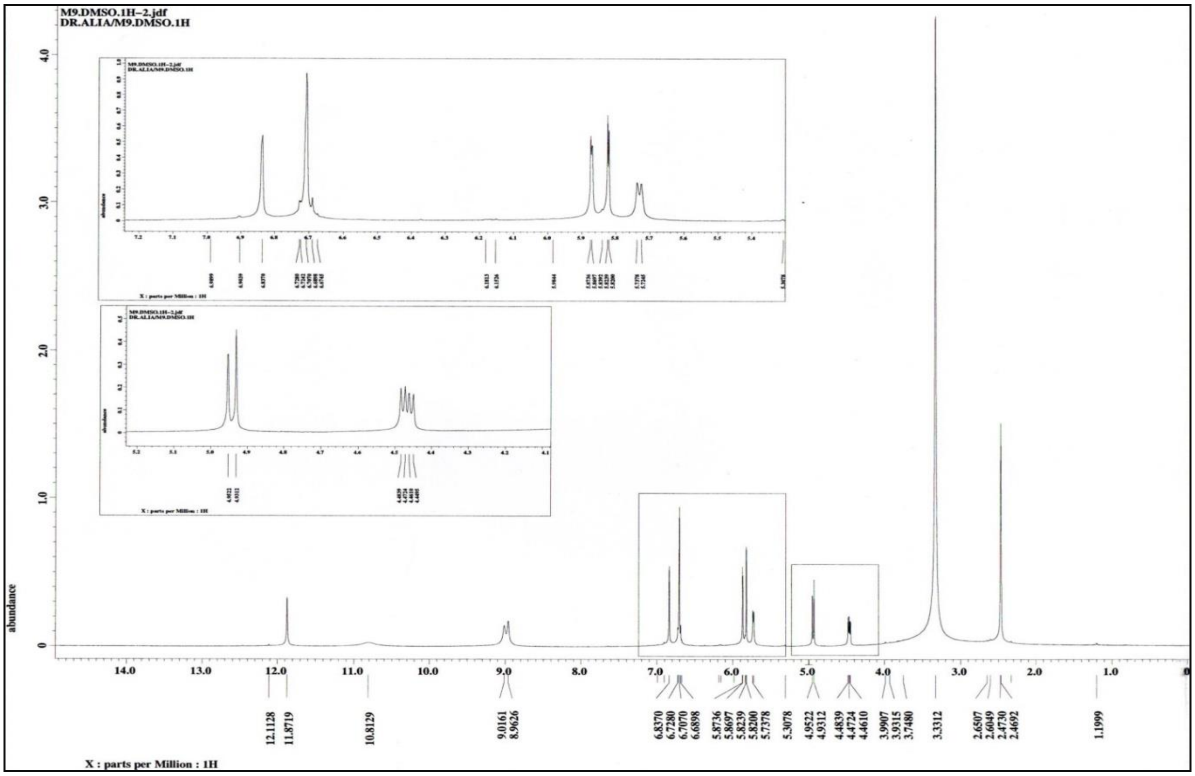

Figure S1. ^1H NMR spectrum of compound (**1**), in dmso-*d6* (500 MHz).

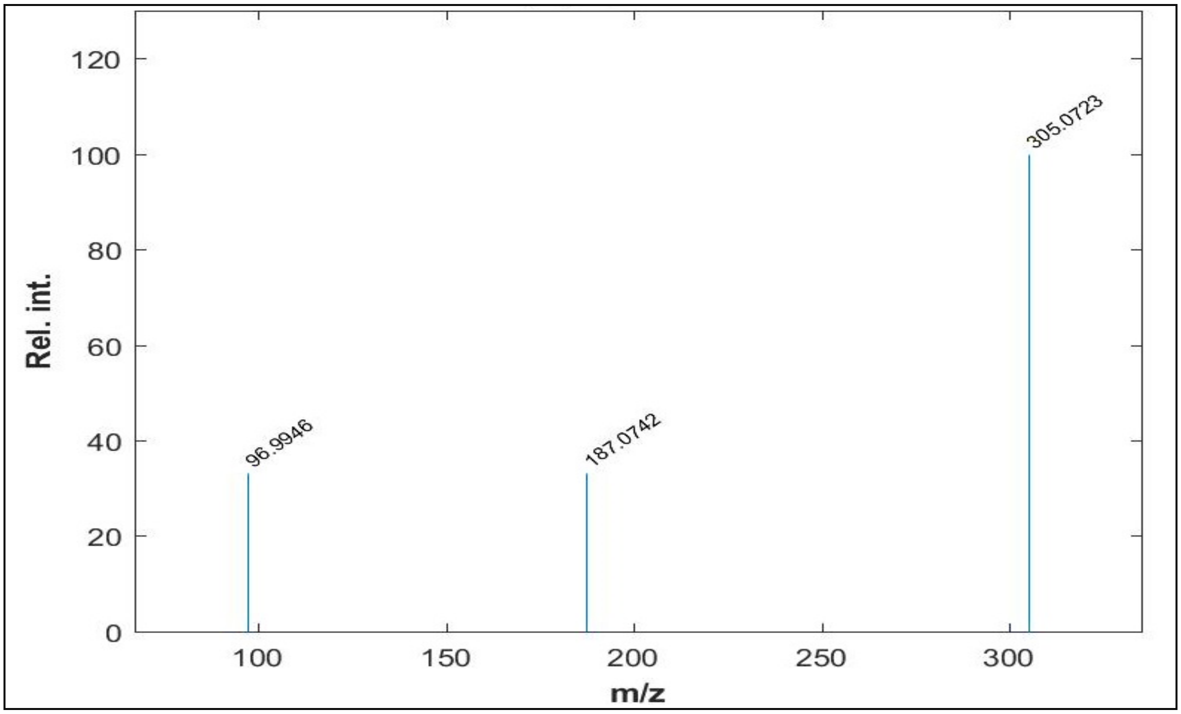

Figure S2. Positive HRMS spectrum of compound (**1**).

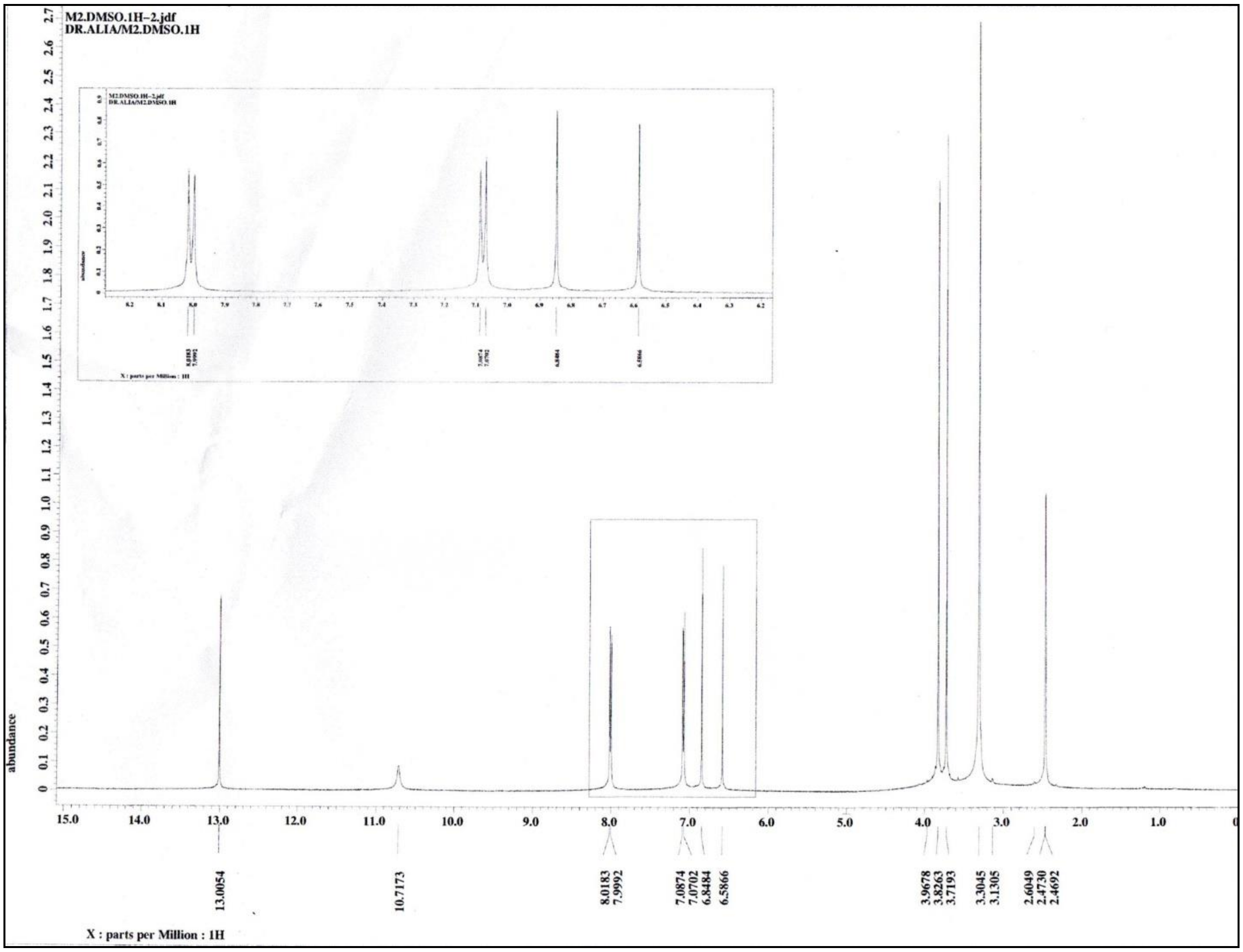

Figure S3. ^{1}H NMR spectrum of compound (**2**), in dmso-*d6* (500 MHz).

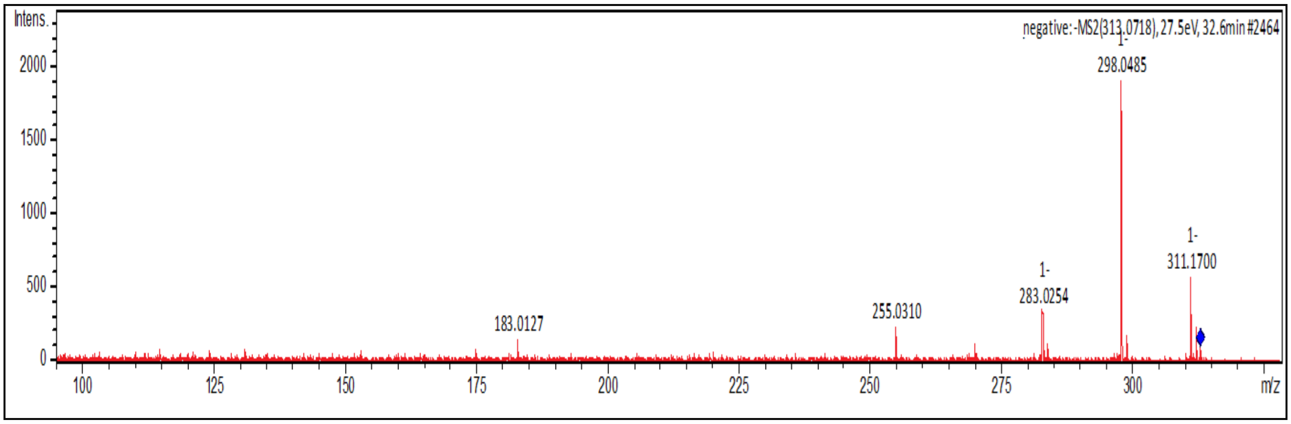

Figure S4. Negative HRMS spectrum of compound (**2**).

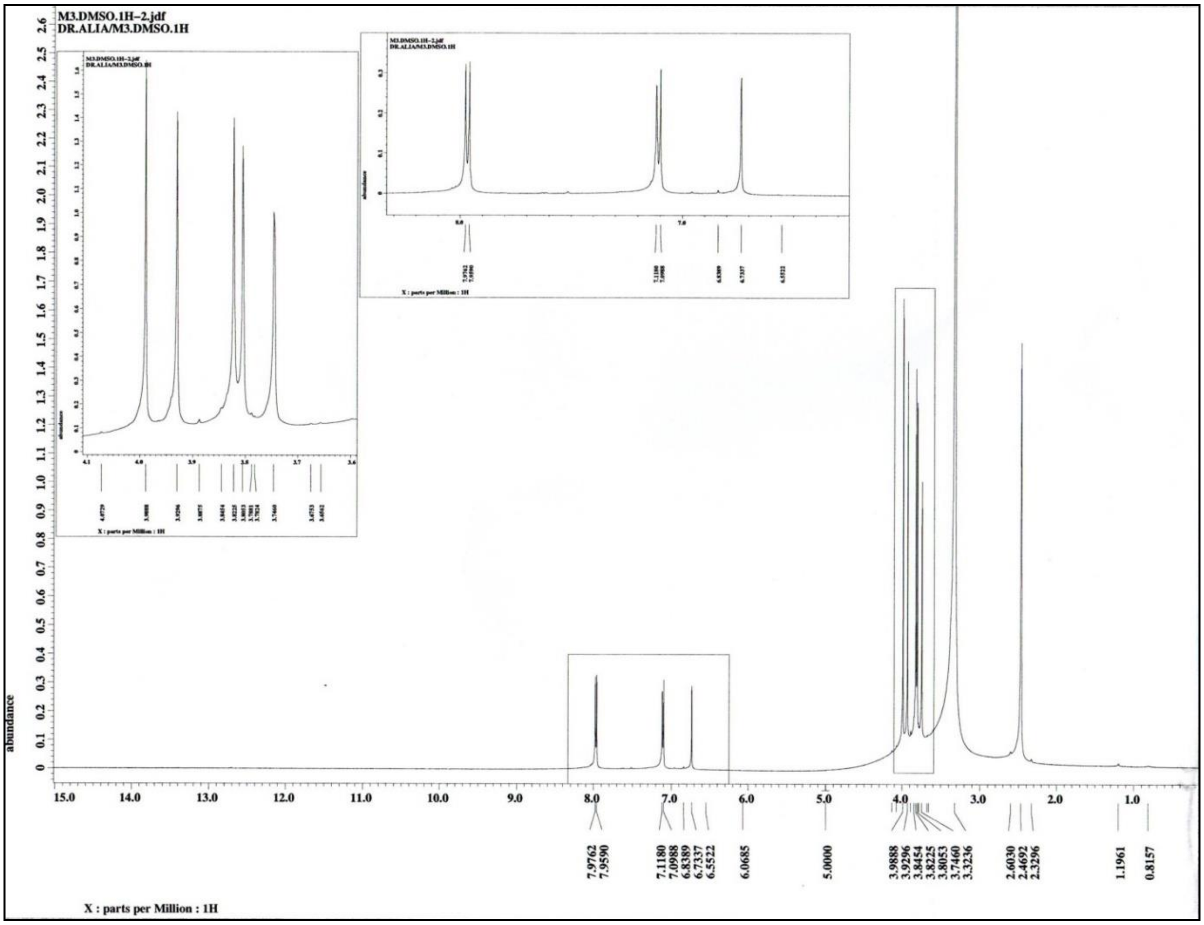

Figure S5. ^{1}H NMR spectrum of compound (**3**), in dmso-*d6* (500 MHz).

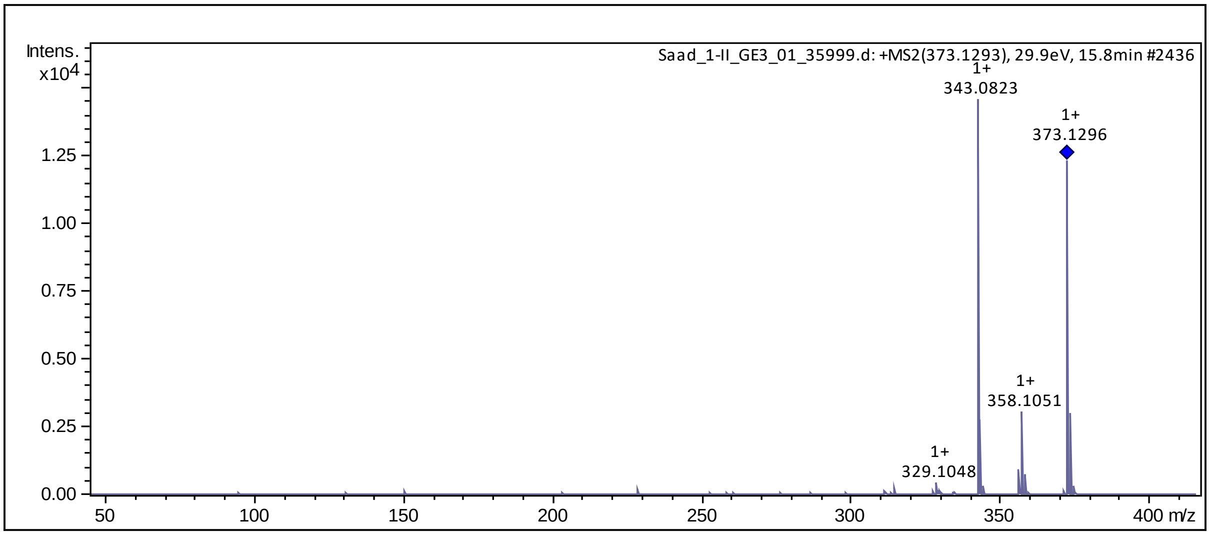

Figure S6. Positive HRMS spectrum of compound (**3**).

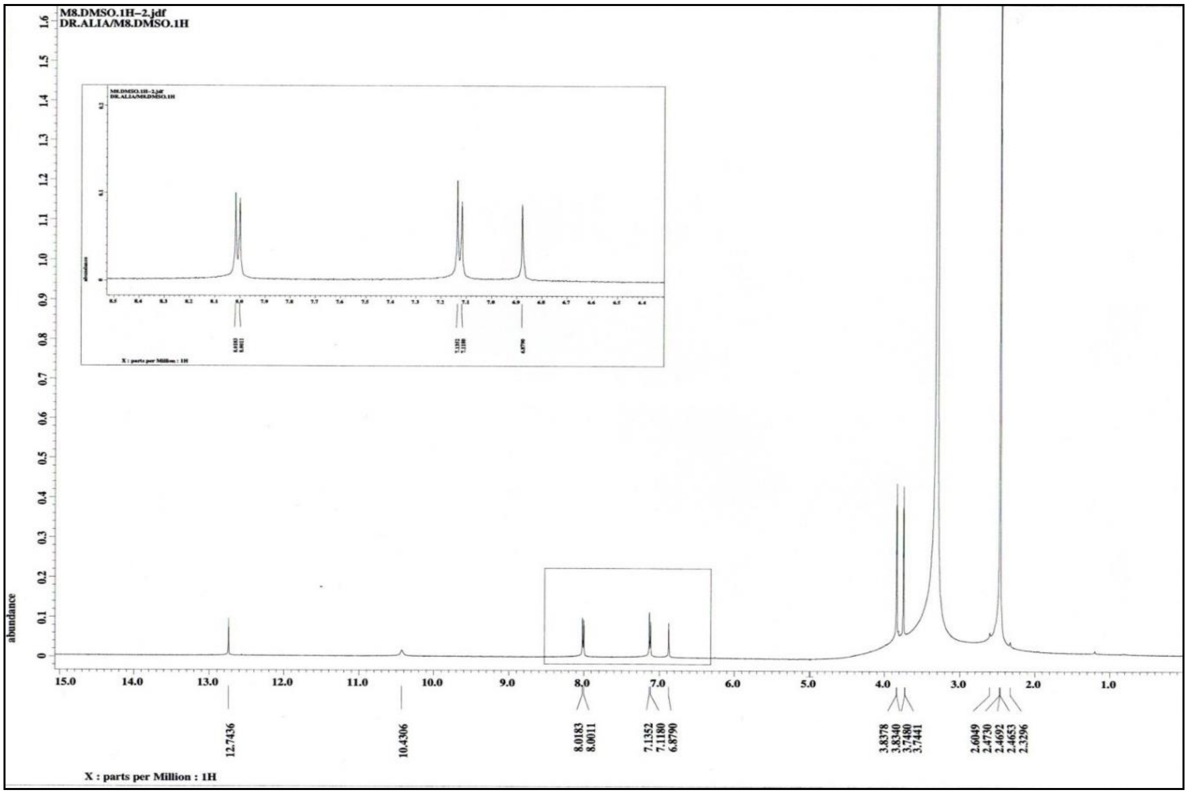

Figure S7. ^{1}H NMR spectrum of compound (**4**), in dmso-*d6* (500 MHz).

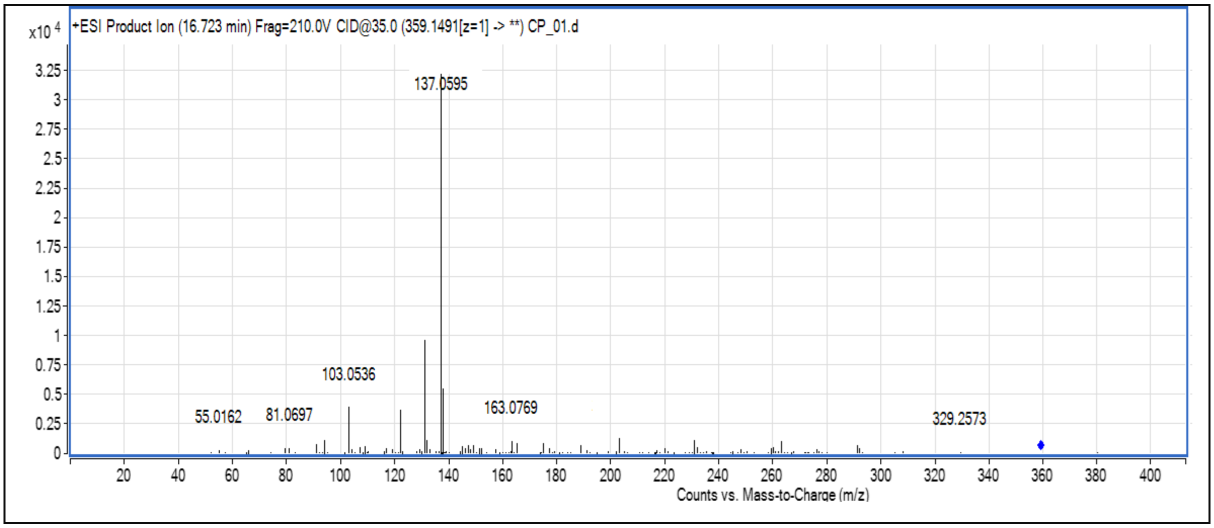

Figure S8. Positive HRMS spectrum of compound (**4**).

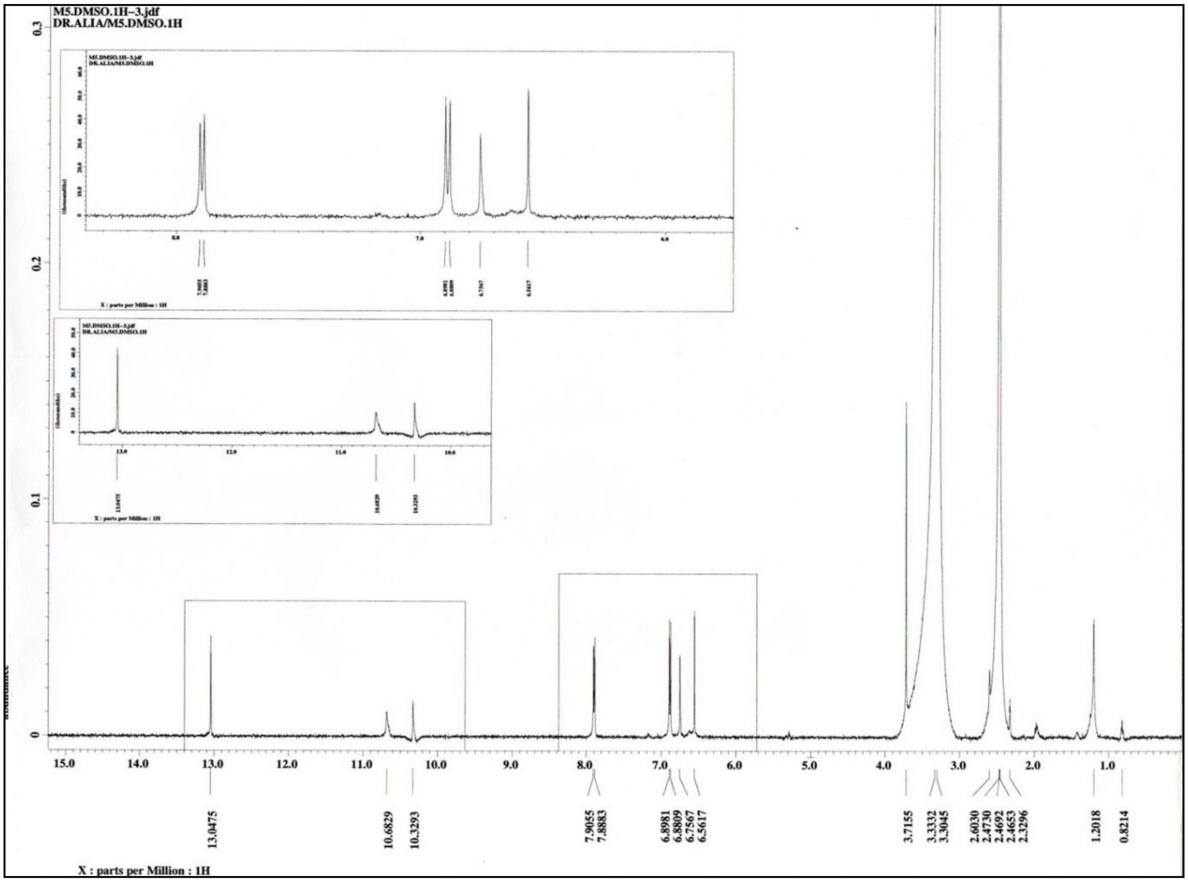

Figure S9. ^{1}H NMR spectrum of compound (**5**), in dmso-*d6* (500 MHz).

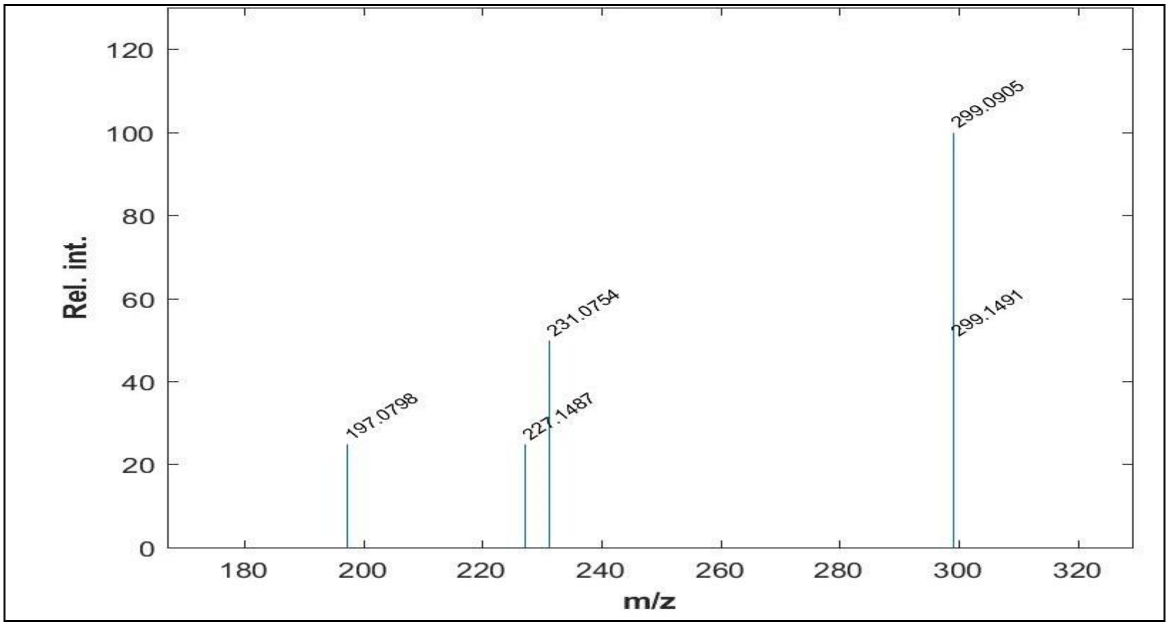

Figure S10. Negative HRMS spectrum of compound (**5**)

Figure S11. 2D pictures showing the receptor interactions and positioning between the docked α-ketoamide inhibitor (**KI**) besides the examined five flavonoids (**1-5**) inside the binding site of SARS-CoV-2 Mpro.

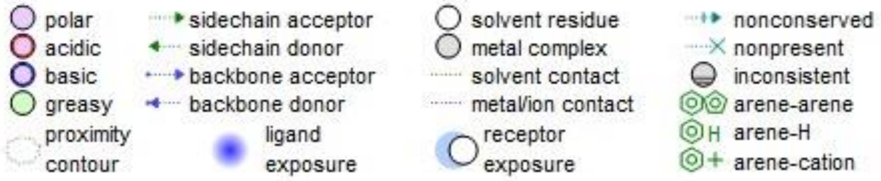

No.	Compound	2D binding interactions
**KI**	**α-ketoamide inhibitor**	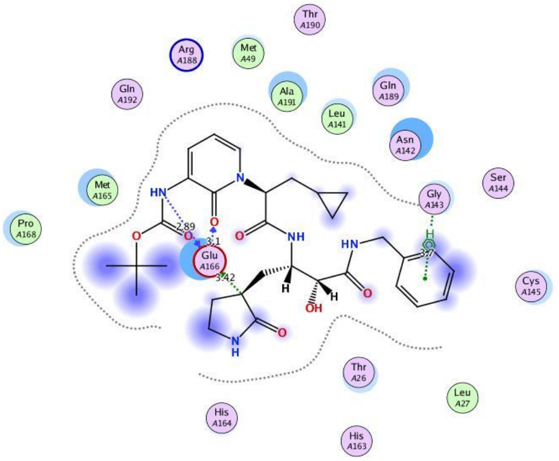
**1**	**Taxifolin**	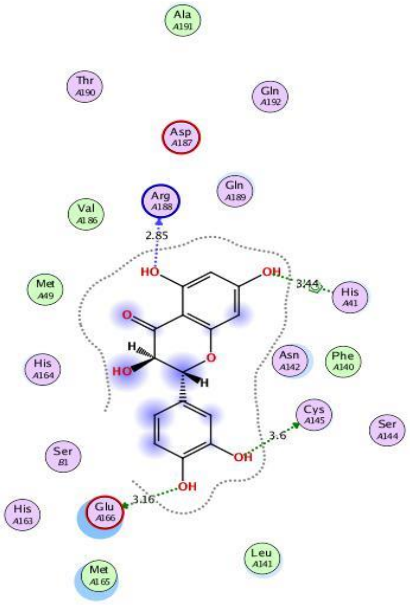

2	**Pectolinargenin**	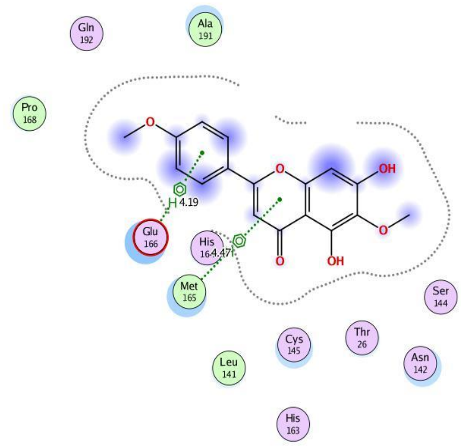
3	**Tangeretin**	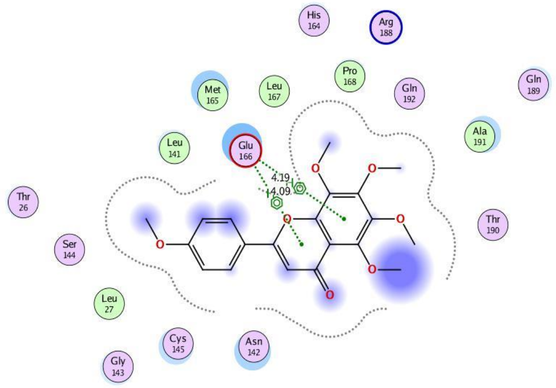
4	**Gardenin B**	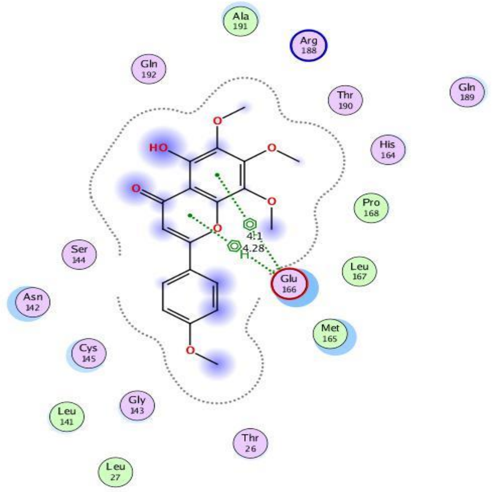

<table>
<tr><td>5</td><td>Hispidulin</td><td>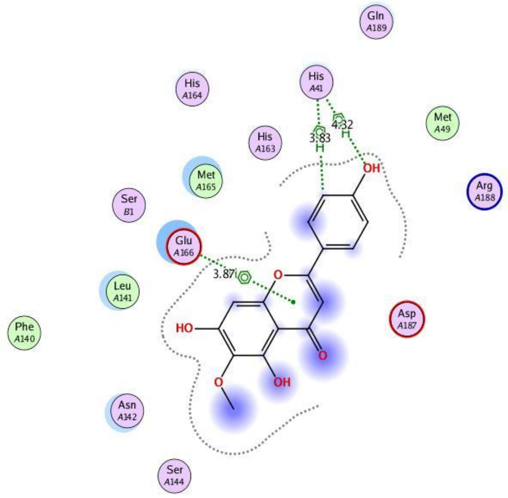
</td></tr>
</table>
